# The Association Between Hamstring Tightness and Chronic Low Back Pain: A Comparative Study

**DOI:** 10.7759/cureus.106169

**Published:** 2026-03-30

**Authors:** Amit Hegde, Atul Sareen, Ravi Shankar, Joydeep Das, Naveen Gowda, Praveen Shetty, Abhishek Sengupta, Rajesh K Chopra

**Affiliations:** 1 Orthopaedics, Safdarjung Hospital, New Delhi, IND; 2 Orthopaedics, Vardhman Mahavir Medical College and Safdarjung Hospital, New Delhi, IND; 3 Orthopaedics and Trauma, Safdarjung Hospital, New Delhi, IND

**Keywords:** active knee extension test(ake), bmi, hamstring flexibility, height, lumbar lordosis, passive straight leg raising test(pslrt)

## Abstract

Introduction

Chronic low back pain (CLBP) is defined as pain in the lower back that persists for more than three months. It is a common musculoskeletal condition and a major contributor to functional impairment and social limitations among young adults. Hamstring flexibility refers to the ability of the hamstring muscles to stretch to their full length. Since both the hamstrings and lumbar extensor muscles originate from the pelvis, a biomechanical relationship may exist between them. Decreased flexibility or weakness in these muscles may contribute to the development of low back pain. Tight hamstrings, which may result from previous injury or insufficient physical activity, can restrict pelvic mobility and limit movement of the lumbar spine. Such limitations may be associated with repetitive microtrauma, thereby contributing to the development of back pain. This study aimed to examine the association between hamstring tightness and CLBP.

Materials and methods

A cross-sectional analytical study was performed after obtaining ethical approval. The study included 40 patients diagnosed with CLBP and 40 age-matched healthy individuals who served as controls. Hamstring flexibility was evaluated using the active knee extension (AKE) test and the passive straight leg raising test (PSLRT), conducted on multiple occasions. Anthropometric parameters, including height, weight, and BMI, were also recorded. Statistical analysis of the collected data was carried out using the chi-square test and correlation analysis. The analysis was performed using SPSS version XX (IBM Corp., Armonk, NY). A p-value < 0.05 was considered statistically significant (α = 0.05). Pearson’s correlation coefficient was used for normally distributed variables.

Results

Abnormal findings in the PSLRT were observed in 35 (87.5%) cases on the right side and 32 (80%) cases on the left side. In contrast, among the control group, only five (12.5%) individuals showed abnormal SLRT results on both sides. These differences were statistically significant (right: χ² = 45.00, p < 0.001; left: χ² = 36.10, p < 0.001). The AKE test revealed hamstring tightness on the right side in 35 (87.5%) cases compared to five (12.5%) controls, and on the left side in 33 (82.5%) cases compared to seven (17.5%) controls. These differences were also statistically significant (p < 0.001). The mean age of participants in the case group was 28.50 ± 5.48 years, while the control group had a mean age of 28.28 ± 5.11 years. Most participants were within the 20-40 year age range, a group in which mechanical low back pain is commonly seen. Both the AKE test and PSLRT indicated significantly reduced hamstring flexibility among individuals with CLBP compared to the control group. In addition, a positive correlation was identified between increased height and the presence of hamstring tightness.

Conclusions

The findings of this study suggest that individuals suffering from CLBP have significantly decreased hamstring flexibility compared to healthy individuals. The study also demonstrated an association between increased height and greater hamstring tightness in both case and control groups.

## Introduction

Several factors are associated with the presence of low back pain, including increased lumbar lordosis, weakened abdominal muscles, decreased endurance and flexibility of back extensor muscles, and shortened iliopsoas and hamstring muscles. Changes in body composition and other related factors may also play a role. Furthermore, reduced spinal mobility resulting from impaired muscle synergy can increase the energy demand required to maintain posture [1]. According to the World Health Organization, chronic primary low back pain refers to pain that persists or recurs for more than three months in the absence of any identifiable structural abnormality, disease, or deformity. Chronic low back pain (CLBP) is a prevalent condition, affecting roughly one in six adults worldwide, and is often manageable through non-surgical approaches in primary and community healthcare settings [2].

CLBP is defined as back pain lasting for more than three months [3]. It is one of the major causes of functional and social limitations in the young population and affects approximately 15-45% of the general population. Additionally, 50-80% of adults experience at least one episode of low back pain during their lifetime [4]. Although symptoms often subside with analgesics and muscle relaxants, allowing individuals to resume normal activities, a subset of patients may progress to chronic pain and disability. Various factors, such as rapid growth spurts, altered lumbar lordosis, poor endurance of superficial abdominal and back extensor muscles, and tightness of back extensor and hamstring muscles, may be associated with low back pain [3].

The hamstring muscle group consists of the semitendinosus, semimembranosus, and biceps femoris. These muscles originate from the inferomedial impression on the upper part of the ischial tuberosity and insert into the posterior surface of the tibia. Hamstring flexibility refers to the ability of these muscles to lengthen to their full range [5]. Since both the hamstrings and lumbar extensors share a common pelvic origin, dysfunction in either group may influence the other and may be associated with the development of low back pain. Hamstring tightness, which may result from previous injury or physical inactivity, can reduce pelvic mobility and lumbar motion and may contribute to repetitive microtrauma. It may also be associated with thoracic kyphosis, spondylosis, and intervertebral disc degeneration [6].

Muscle imbalances can lead to habitual overuse of specific joints and faulty movement patterns, resulting in microtrauma, dysfunction, and chronic injury. Abnormal postures increase shear and compressive forces on joints, placing excessive stress on articular surfaces and contributing to mechanical low back pain. Inadequate hamstring flexibility may impair the maintenance of normal spinal curvature, particularly in sitting positions, leading to increased lumbar flexion and subsequent pain. Additionally, hamstring shortening and spasm can cause posterior pelvic tilt, thereby reducing pelvic mobility and increasing lumbar stress during functional activities [6].

Low back pain in younger individuals is predominantly mechanical in origin, whereas degenerative changes are more common in older populations. In younger individuals, it significantly impacts work efficiency and imposes an economic burden, often leading to overuse of nonsteroidal anti-inflammatory drugs (NSAIDs). Fear of pain may discourage physical activity, which can contribute to hamstring tightness due to inactivity. Although patients are frequently advised to perform back and hamstring stretching exercises, it is important to note that even asymptomatic individuals may exhibit hamstring tightness. Due to diagnostic uncertainty, especially in cases of mechanical low back pain, patients are often subjected to unnecessary imaging, such as MRI and CT scans, even in the absence of significant structural pathology.

## Materials and methods

Study design and ethical approval

This cross-sectional analytical study was conducted at the Central Institute of Orthopedics, Vardhman Mahavir Medical College and Safdarjung Hospital, New Delhi. Ethical approval was obtained from the Institutional Ethics Committee, Vardhman Mahavir Medical College and Safdarjung Hospital (IEC/VMMC/SJH/Thesis/2020-11/CC-194).

Study sample

All patients with low back pain aged 20 to 45 years who were not receiving any form of treatment or physiotherapy were included in the study. Age- and sex-matched healthy individuals were recruited as controls. Patients with a history of spinal surgery, congenital spinal deformities, hip or knee deformities, or prior hip or knee surgery were excluded. Additionally, individuals presenting with radiating back pain to the lower limbs, lower extremity neurovascular pathology, hyperlaxity, connective tissue disorders, or traumatic spinal injury were excluded. Patients undergoing treatment or physiotherapy were excluded to avoid potential confounding effects on hamstring flexibility measurements; however, this may limit the external validity of the findings.

Sample size determination

The sample size of 80 participants (40 cases and 40 controls) was determined based on feasibility within the study period. A post hoc power analysis based on observed effect sizes (Cohen’s d > 0.8) indicated that the study had sufficient statistical power (power > 80%) to detect differences in hamstring flexibility between groups.

Following the initial assessment, participants were categorized into case and control groups. Hamstring flexibility was assessed using the active knee extension (AKE) test and the passive straight leg raising test (PSLRT), performed on three separate occasions under identical examination conditions. The validity of the AKE test is based on the anatomical insertions of the hamstring muscle group, and its convergent validity has been demonstrated through a strong correlation with the active SLRT [7].

Active knee extension (AKE) test

**Figure 1:**
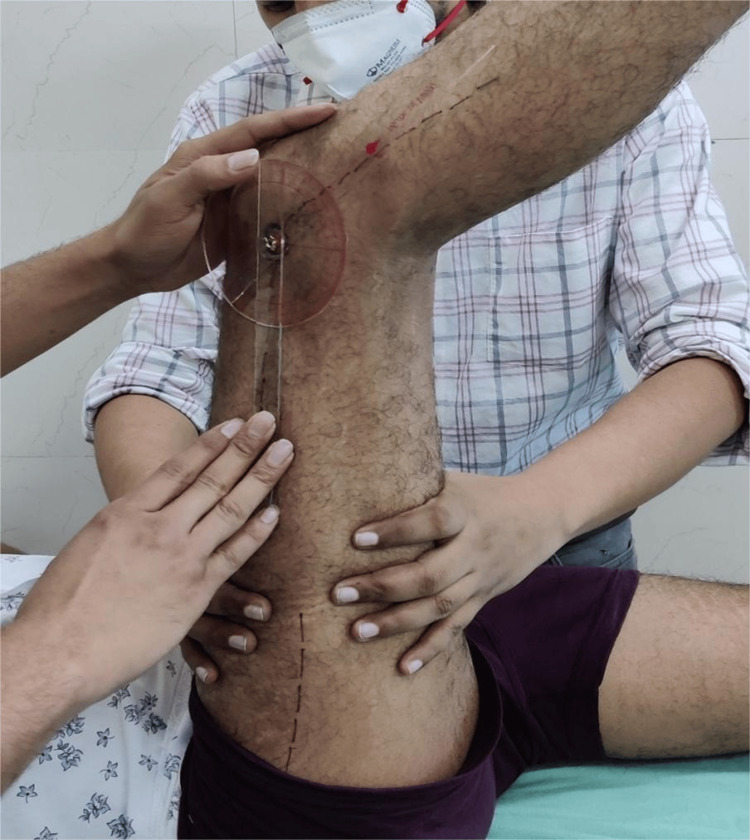
Active knee extension test Picture clicked by the author after obtaining consent

After obtaining informed consent, hamstring flexibility was evaluated using the AKE test (popliteal angle test) with a universal goniometer (Figure 1). The participant was positioned supine on an examination table, with the contralateral limb fully extended to minimize pelvic movement. Anatomical landmarks, including the greater trochanter, lateral epicondyle of the femur, and lateral malleolus, were identified and marked. For assessment of the right lower limb, the left lower limb remained extended on the table. The participant actively flexed the hip to 90° with the knee initially maintained in 90° of flexion. From this position, the participant was instructed to gradually extend the knee until a mild stretch was felt in the posterior thigh, while keeping the foot relaxed. The examiner stabilized the thigh throughout the maneuver [6,7].

A universal goniometer was positioned along the lateral aspect of the knee joint. The fulcrum was placed over the lateral epicondyle of the femur, the proximal arm was aligned with the long axis of the femur using the greater trochanter as a reference, and the distal arm was aligned with the long axis of the leg using the lateral malleolus as a reference [7]. The participant extended the knee to the maximum possible range until a mild stretch was perceived. The angle between the femur and tibia was recorded, and this value was subtracted from 180° to calculate the AKE angle. An AKE angle of less than 20° was considered normal based on previously validated cutoff values described in the literature [8,9]. The test was performed three times on three separate days within one week by the same clinician, and the average of the three readings was recorded as the final AKE value.

Passive straight leg raising test

**Figure 2:**
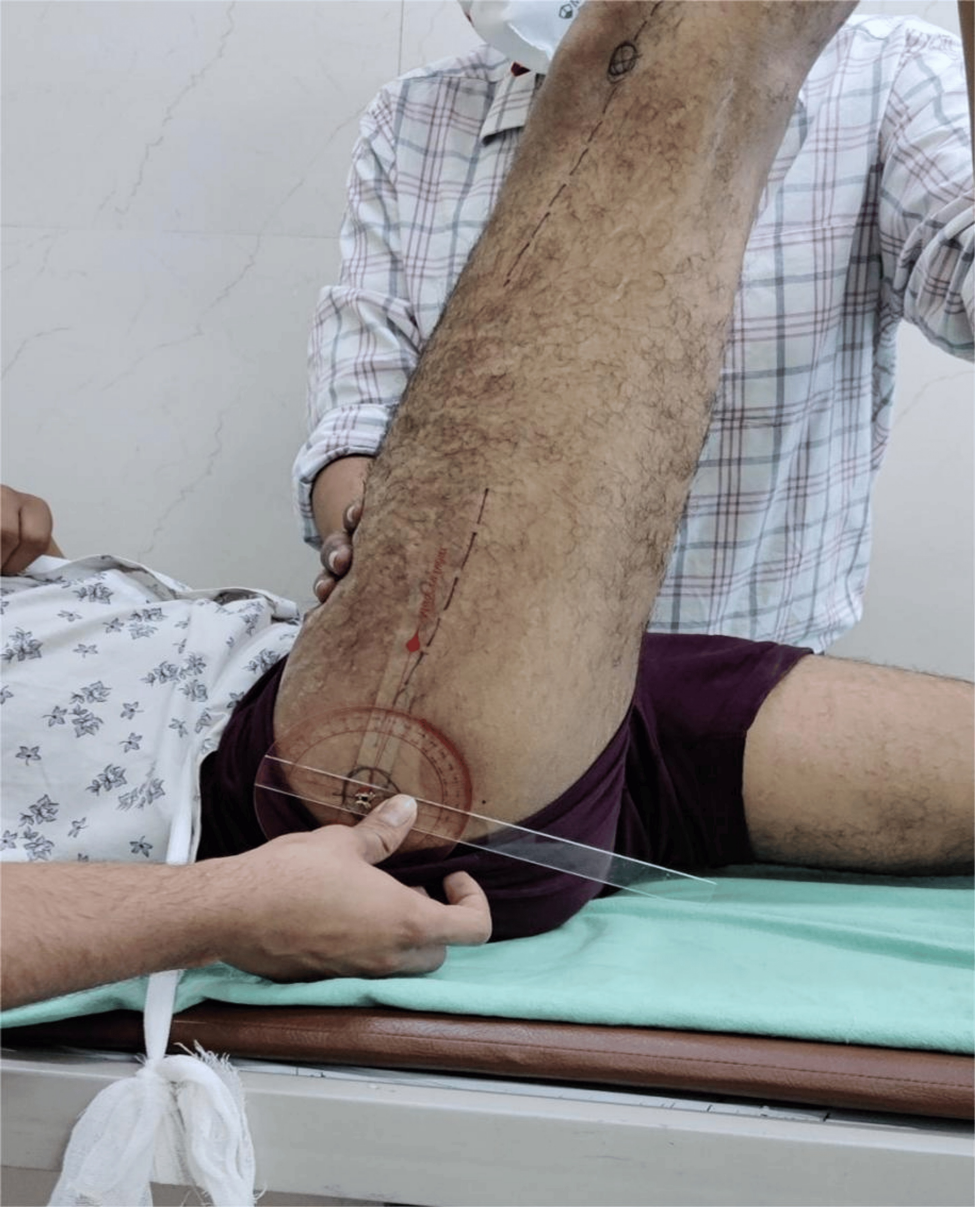
Passive straight leg raising test Picture taken by the author after obtaining consent

The participant was positioned supine on an examination couch with both lower limbs extended, ensuring careful monitoring of pelvic rotation throughout the assessment. The clinician passively raised the participant’s leg with the knee maintained in full extension, flexing the hip until the onset of discomfort. An assistant stabilized the contralateral lower limb in an extended position to prevent compensatory movements [10]. The range of motion (ROM) was measured using a goniometer, with its axis placed over the tip of the greater trochanter. The proximal arm of the goniometer was aligned along the long axis of the raised lower limb, while the distal arm remained parallel to the couch during measurement. An angle of 80° or greater was considered within the normal range as per established normative values (Figure 2) [9-12]. The test was performed three times on three separate days within one week by the same clinician, and the mean of the three measurements was recorded as the final value.

Participants were asked to stand upright against a wall marked with centimeter graduations for height measurement. A straight metal ruler was placed horizontally at the highest point of the head, ensuring contact with the wall. The participant was then asked to step aside, and the height was recorded using a measuring tape. This procedure was repeated three times, and the average of the three measurements was considered the final height value. Body weight was measured using a manual weighing scale available in the outpatient clinic, with participants standing upright on the machine. The BMI of each participant was calculated.

Outcome measures

Participants were examined in the Orthopaedic Outpatient Department (OPD) and Emergency Department to assess hamstring flexibility using the AKE test and PSLRT. In the AKE test, a knee extension angle greater than 20° was considered abnormal, indicating reduced hamstring flexibility. In PSLRT, a hip flexion angle of less than 80° was regarded as abnormal, suggesting decreased hamstring flexibility. The validity of the AKE test is supported by the anatomical basis of the hamstring muscle group and its insertions. Furthermore, studies comparing the AKE test with PSLRT have demonstrated strong convergent validity, showing a significant correlation between the outcomes of the two assessment methods [9,11,12]. All measurements were performed by a single examiner to minimize inter-observer variability. Previous studies have demonstrated high intra-rater reliability for both AKE and SLRT (intraclass correlation coefficient: > 0.90) [11,13].

Statistical analysis

Statistical analysis was performed using SPSS version XX (IBM Corp., Armonk, NY). A p-value < 0.05 was considered statistically significant (α = 0.05). The normality of data was assessed using the Shapiro-Wilk test. Continuous variables were compared using the independent (unpaired) t-test, while categorical variables were analyzed using the Chi-square test. Effect sizes were calculated using the Phi coefficient (φ), with values greater than 0.5 indicating a large effect size [14]. Pearson’s correlation coefficient was used to evaluate the relationship between anthropometric variables (height, weight, and BMI) and hamstring flexibility. Linear regression analysis was performed, with assumptions including linearity, normality of residuals, homoscedasticity, and absence of multicollinearity assessed and confirmed.

## Results

An independent (unpaired) t-test was used to compare the mean demographic parameters between the two groups, as shown in Table 1. The mean age of the case group was 28.50 ± 5.48 years, while the control group had a mean age of 28.28 ± 5.11 years, and this difference was not statistically significant (p = 0.85).

**Table 1:** Anthropometric details An unpaired t-test was used to compare these observations SD: standard deviation; BMI: body mass index

	Cases (n = 40), mean ± SD	Controls (n = 40), mean ± SD	P-value
Age, years	28.50 ± 5.48	28.28 ± 5.11	0.85
Height, cm	174.08 ± 7.81	167.40 ± 6.01	< 0.01
Weight, kg	68.13 ± 5.45	64.25 ± 5.74	< 0.01
BMI, kg/m^2^	22.55 ± 1.60	22.91 ± 1.50	0.30

However, statistically significant differences were observed in height and weight between the groups. Cases had a mean height of 174.08 ± 7.81 cm, compared to 167.40 ± 6.01 cm in controls (p < 0.01). Similarly, the mean weight among cases was 68.13 ± 5.45 kg, whereas controls had a mean weight of 64.25 ± 5.74 kg, which was also statistically significant (p < 0.01). The mean BMI was 22.55 ± 1.60 kg/m² in cases and 22.91 ± 1.50 kg/m² in controls, with no statistically significant difference. The magnitude of differences between groups was further assessed using Cohen’s d. A large effect size was observed for height (d = 0.96) and weight (d = 0.69), while BMI demonstrated a small effect size (d = 0.23). According to Cohen’s criteria, d values of 0.2, 0.5, and 0.8 represent small, medium, and large effect sizes, respectively [14].

The distribution of PSLRT findings between cases and controls was analyzed using the chi-square test of independence, as shown in Table 2. On the right side, abnormal SLRT findings were observed in 35 out of 40 cases (87.5%), whereas only five out of 40 controls (12.5%) demonstrated abnormal findings. Conversely, normal SLRT findings were present in five cases (12.5%) and 35 controls (87.5%). The association between group status (case/control) and right-side SLRT findings was found to be statistically significant (χ² = 45.00, df = 1, p < 0.001). The observed phi values (> 0.7) indicate a large effect size, suggesting a strong association between CLBP and hamstring tightness. According to Cohen’s criteria, phi (φ) values of 0.1, 0.3, and 0.5 represent small, medium, and large effect sizes, respectively [14].

**Table 2:** Comparison of passive SLRT between both groups A Chi-square (χ²) test was used to compare the distribution of SLRT results between the two groups. The degree of freedom (df) for the test was 1, calculated as (rows−1 × columns−1) SLRT: straight leg raising test

	Cases (n = 40)		Controls (n = 40)		P-value
	N	%	N	%	
SLRT right side					
Abnormal	35	87.5	5	12.5	< 0.001
Normal	5	12.5	35	87.5	
SLRT left side					
Abnormal	32	80.0	5	12.5	< 0.001
Normal	8	20.0	35		

Similarly, on the left side, abnormal SLRT was present in 32 cases (80%) and five controls (12.5%), while normal SLRT was observed in eight cases (20%) and 35 controls (87.5%). The difference between the groups was again statistically significant (χ² = 36.10, df = 1, p < 0.001). The effect size (φ = 0.67) also indicated a large association between CLBP and abnormal SLRT findings on the left side. Overall, the results demonstrate that abnormal passive SLRT was significantly more prevalent in patients with CLBP compared to normal individuals, with large effect sizes indicating a strong association between hamstring tightness and CLBP.

As depicted in Table 3, the association between CLBP (cases) and hamstring tightness measured using the AKE test was analyzed using the chi-square test of independence. On the right side, 87.5% (35/40) of the cases showed hamstring tightness, whereas only 12.5% (5/40) of the control group demonstrated hamstring tightness. Similarly, on the left side, 82.5% (33/40) of the cases showed hamstring tightness, while only 7.5% (3/40) of the controls had hamstring tightness. In contrast, the majority of the control group showed normal AKE test findings on both sides (87.5% on the right side and 92.5% on the left side) compared with a much smaller proportion of cases (12.5% on the right side and 17.5% on the left side).

**Table 3:** Comparison of the AKE test between the groups The Chi-square test was used to compare the prevalence of hamstring tightness between the case and control groups. The degree of freedom (df) for this analysis was 1 AKE: active knee extension

	Cases (n = 40)		Controls (n = 40)		P-value
	N	%	N	%	
AKE right side					
Normal	5	12.5	35	87.5	< 0.001
Hamstring tightness	35	87.5	5	12.5	
AKE left side					
Normal	7	17.5	37	92.5	< 0.001
Hamstring tightness	33	82.5	3		

The Chi-square test demonstrated a highly statistically significant association between study group and AKE test findings (p < 0.001) with degrees of freedom (df = 1) for both sides, indicating that the distribution of hamstring tightness differs significantly between cases and controls. The effect size, calculated using the Phi coefficient (φ), was approximately 0.75 for the right side and 0.77 for the left side, indicating a large effect size. This suggests a strong association between CLBP and hamstring tightness as measured by the AKE test. Overall, the findings indicate that hamstring tightness assessed by KEA is significantly more prevalent in patients with CLBP compared to normal individuals, demonstrating a strong relationship between reduced hamstring flexibility and chronic low back pain.

Table 4 shows the association between height, weight, and BMI with SLRT and AKE. Pearson’s correlation analysis was performed to evaluate the relationship between anthropometric variables (height, weight, and BMI) and hamstring flexibility measured using the AKE test and SLRT. Confidence intervals (95% CI) for correlation coefficients were calculated to assess the precision of the estimates.

**Table 4:** Correlation of weight, height, and BMI with the PSLRT and AKE test (n = 80) The Spearman rank correlation coefficient (r) was used to evaluate the relationship between anthropometric parameters and hamstring flexibility BMI: body mass index; PSLRT: passive straight leg raising test; AKE: active knee extension

		Height	Weight	BMI
KEA (R)	r value	0.341	0.240	-0.121
	P-value	0.002	0.032	0.284
	N	80	80	80
KEA (L)	r value	0.335	0.234	-0.131
	P-value	0.002	0.037	0.246
	N	80	80	80
SLR (R)	r value	-0.353	-0.245	0.109
	P-value	0.001	0.028	0.337
	N	80	80	80
SLR (L)	r value	-0.276	-0.221	0.065
	P-value	0.013	0.049	0.565
	N	80	80	80

For the AKE test, height demonstrated a moderate positive correlation on both the right (r = 0.341, 95% CI: 0.13 to 0.52, p = 0.002) and left sides (r = 0.335, 95% CI: 0.12 to 0.51, p = 0.002). Weight showed a weak but statistically significant positive correlation with AKE values (right: r = 0.240, 95% CI: 0.02 to 0.44, p = 0.032; left: r = 0.234, 95% CI: 0.01 to 0.43, p = 0.037). In contrast, BMI demonstrated a weak negative correlation that was not statistically significant (right: r = -0.121, 95% CI: -0.33 to 0.11, p = 0.284; left: r = -0.131, 95% CI: -0.34 to 0.09, p = 0.246).

For SLRT, height showed a moderate negative correlation on the right side (r = -0.353, 95% CI: -0.53 to -0.14, p = 0.001) and a weaker but significant negative correlation on the left side (r = -0.276, 95% CI: -0.47 to -0.05, p = 0.013). Weight demonstrated a weak negative correlation with SLRT values (right: r = -0.245, 95% CI: -0.44 to -0.03, p = 0.028; left: r = -0.221, 95% CI: -0.42 to -0.01, p = 0.049). BMI showed no significant correlation with SLRT values (right: r = 0.109, 95% CI: -0.12 to 0.32, p = 0.337; left: r = 0.065, 95% CI: -0.17 to 0.29, p = 0.565).

Although statistically significant, the observed correlations between anthropometric parameters and hamstring flexibility were modest (r ≈ 0.2-0.35), indicating that these variables explain only a limited proportion of the variability in flexibility. Clinically, this suggests that hamstring tightness is only one component of a complex interplay of biomechanical and neuromuscular factors contributing to chronic low back pain. Therefore, rehabilitation strategies should incorporate a comprehensive approach, including core strengthening, postural correction, and activity modification rather than focusing exclusively on hamstring stretching.

To further explore these relationships, simple linear regression analysis was performed, as shown in Table 5. Before regression analysis, the assumptions of linearity, normality of residuals, homoscedasticity, and absence of multicollinearity were assessed using standard diagnostic methods and were found to be satisfied. Height emerged as a significant predictor of hamstring flexibility for both AKE and SLRT measurements. For AKE, increasing height was associated with an increase in knee extension angle, whereas for SLRT, increasing height predicted a reduction in hip flexion angle. Weight also showed a significant but weaker predictive effect on both AKE and SLRT values. However, BMI did not significantly predict hamstring flexibility in either test (p > 0.05).

**Table 5:** Regression analysis Linear regression analysis of height, weight, and BMI with AKE and PSLRT BMI: body mass index; AKE: active knee extension; PSLRT: passive straight leg raising test

Variable	AKE_R_beta	AKE_R_p	AKE_L_beta	AKE_L_p	SLR_R_beta	SLR_R_p	SLR_L_beta	SLR_L_p
Height	0.341	0.002	0.335	0.002	-0.353	0.001	-0.276	0.013
Weight	0.24	0.032	0.234	0.037	-0.245	0.028	-0.221	0.049
BMI	-0.121	0.284	-0.131	0.246	0.109	0.337	0.065	0.565

The coefficient of determination (R²) values indicated that anthropometric variables explained a small to moderate proportion of the variance in hamstring flexibility (approximately 5-12%), suggesting that additional biomechanical and neuromuscular factors likely contribute to flexibility outcomes.

## Discussion

The present study was conducted to evaluate hamstring flexibility in patients with CLBP and to compare it with that of healthy individuals. Hamstring flexibility was assessed using the AKE test and PSLRT, and its relationship with anthropometric parameters such as height, weight, and BMI was also analyzed. Gajdosik et al. compared SLR performed with stabilization straps, SLR with a flat low back, the AKE test, and the passive knee extension test in measuring hamstring length and flexibility, and concluded that there are significant relationships among all four tests, suggesting that they represent similar, indirect measurements of hamstring length. Therefore, both the PSLRT and AKE tests can be used as reliable tools for assessing hamstring flexibility [13].

In the present study, the mean age of cases was 28.50 ± 5.48 years, while that of controls was 28.28 ± 5.11 years, with no statistically significant difference between the groups. Most participants belonged to the 20-40-year age group, which is commonly affected by mechanical low back pain. This finding is consistent with previous literature reporting a higher prevalence of low back pain among young and middle-aged adults due to increased physical activity, occupational stress, and lifestyle-related factors. The comparison of anthropometric parameters revealed that cases had significantly greater height and weight compared to controls, whereas BMI was comparable between the two groups. These findings suggest that height and weight may be associated with hamstring flexibility and spinal biomechanics, potentially contributing to the presence of low back pain.

The AKE test demonstrated a significantly higher prevalence of hamstring tightness among patients with CLBP. On the right side, 87.5% of cases showed hamstring tightness compared to 12.5% of controls, while on the left side, 82.5% of cases and 7.5% of controls demonstrated hamstring tightness. These differences were highly statistically significant (p < 0.001), supporting the association between hamstring tightness and CLBP. Similar findings were reported by Mistry et al., where the mean AKE values were significantly higher in cases (31.63° ± 8.34°) compared to controls (14.30° ± 9.70°), indicating reduced hamstring flexibility in patients with low back pain [6]. Comparable results were also observed by Jayawardana, who reported lower hamstring flexibility in patients with chronic low back pain compared to healthy individuals using AKE measurements [3].

In contrast to the present findings, some studies have reported no significant association between hamstring tightness and low back pain. For instance, Radwan et al. observed that hamstring tightness alone may not be a definitive contributing factor to low back pain, suggesting that the relationship is multifactorial and influenced by additional biomechanical and functional variables such as core stability, pelvic alignment, and activity levels. However, differences in study design, sample characteristics, and assessment methods may account for these variations. Unlike the present study, which focused on a relatively homogeneous young adult population, their study included a broader demographic range and did not exclusively assess mechanical low back pain, which may have diluted the observed association [15].

Assessment using the PSLRT also revealed a significantly higher prevalence of abnormal findings in cases compared to controls. In the present study, 87.5% of cases demonstrated abnormal SLRT on the right side and 80% on the left side, whereas only 12.5% of controls showed abnormal findings on both sides. These differences were highly statistically significant (p < 0.001), indicating a strong association between reduced hamstring flexibility and CLBP. However, Stutchfield and Coleman reported no association between hamstring flexibility and low back pain in a study conducted among young rowers, although their study lacked a control group and was limited to a specific athletic population [16].

The relationship between anthropometric variables and hamstring flexibility was further analyzed using correlation analysis. A significant positive correlation was observed between AKE angle and height, suggesting that taller individuals exhibited greater hamstring tightness. Conversely, a significant negative correlation was found between SLRT angle and height, indicating reduced hamstring flexibility with increasing height. Similar trends were observed with body weight. However, BMI did not demonstrate any statistically significant correlation with hamstring flexibility in either AKE or SLRT assessments. Sareen et al. similarly observed that individuals with greater height exhibited increased hamstring tightness, while weight and BMI did not show a significant association [17].

The association between hamstring tightness and low back pain can be explained by the biomechanical linkage between the hamstrings and the pelvis. The hamstrings originate from the ischial tuberosity, and increased tightness can restrict pelvic mobility and alter lumbar spine movement. This may disrupt normal lumbopelvic rhythm, increasing mechanical stress on the lumbar spine and surrounding structures, thereby contributing to repetitive microtrauma and persistence of low back pain [18]. The findings of the present study are consistent with several previous studies demonstrating an association between reduced hamstring flexibility and CLBP. These results emphasize that hamstring tightness may contribute to lumbar dysfunction and mechanical back pain. Therefore, assessment of hamstring flexibility should be an integral component of clinical evaluation in patients presenting with low back pain. The present study also highlights the importance of incorporating hamstring stretching and flexibility exercises into rehabilitation programs for patients with CLBP. Improving hamstring flexibility may help restore normal pelvic alignment and lumbar biomechanics, thereby reducing stress on spinal structures and alleviating symptoms.

Limitations of the study

Despite the significant findings, this study has certain limitations. The sample size was relatively small, which may limit the generalizability of the results. The cross-sectional design precludes establishing a causal relationship between hamstring tightness and CLBP. Additionally, important confounding factors such as physical activity levels, occupational exposure, and duration of symptoms were not assessed, which may influence both hamstring flexibility and the occurrence of low back pain. The absence of blinding during assessment may introduce observer bias. Although standardized measurement techniques were used, the possibility of measurement bias cannot be entirely excluded. Future studies with larger sample sizes and longitudinal designs are needed to better establish causality.

Clinical implications

The findings of this study emphasize the importance of assessing hamstring flexibility in patients presenting with CLBP. Early identification of hamstring tightness may help clinicians design targeted rehabilitation programs, including stretching and strengthening exercises aimed at improving flexibility and restoring normal lumbopelvic mechanics. Incorporating hamstring stretching into physiotherapy protocols may contribute to reducing pain severity and improving functional outcomes.

## Conclusions

The present study was conducted to evaluate hamstring flexibility in individuals with CLBP and to compare it with that of healthy individuals. The findings demonstrated that hamstring flexibility was significantly reduced in patients with CLBP compared to healthy individuals, as assessed by both the AKE test and the PSLRT. A significantly higher proportion of cases exhibited hamstring tightness compared to the control group. In addition, the study revealed a significant association between anthropometric parameters and hamstring flexibility. Height and weight were significantly correlated with the results of both AKE and SLRT tests, indicating that individuals with greater height and weight tended to exhibit increased hamstring tightness, whereas BMI was not significantly associated. These findings suggest that hamstring tightness is linked to CLBP. However, due to the cross-sectional design of the study, causal relationships cannot be established. Further longitudinal studies are required to determine the direction and nature of this relationship.
